# Supplementary material for: High-k Fluoropolymers Dielectrics for Low-Bias Ambipolar Organic Light Emitting Transistors (OLETs)
Source: Materials (Basel). 2021 Dec 11;14(24):7635. doi: 10.3390/ma14247635 (PMC8704791; doi:10.3390/ma14247635)
Supplement: Supplementary file 1 [file materials-14-07635-s001.zip › materials-1485269-supplementary.pdf]

Supporting Information

High-*k* Fluoropolymers Dielectrics for Low-bias Ambipolar Organic Light Emitting Transistors (OLETs)

Ahmed Albeltagi [1,2,¥], Katherine Gallegos-Rosas [2] and Caterina Soldano [2,*,¥]

[1] Department of Physics and Mathematics, Institute of Photonics, University of Eastern Finland, Joensuu Campus, 80100, Joensuu, Finland

[2] Department of Electronics and Nanoengineering, School of Electrical Engineering, Aalto University, Tietotie 3 (Micronova), 02150 Espoo, Finland

[*]Corresponding author

e-mail (CS): caterina.soldano@aalto.fi

[¥] These authors contributed equally

1. Solvent properties

Summary table including several physical properties of solvents used in the work.

Table S1. Properties of solvent used in this work (methylethylketone: MEK, cyclopentanone: CP).

	**Methylethylketone**	**Cyclopentanone**
**density (kg/m^3)**	805	949
**viscosity @RT (mPa/s)**	0.42	1.073
**boiling point (°C)**	80	131
**dielectric constant**	18.51	14.45
**polarity (D)**	2.78	3.58

2. Optical images of substrates

Figure S1 shows the optical image of the representative substrates used in this work. From left to right: ITO on glass, PMMA on ITO on glass and P(VDF-TrFE-CFE:CP) on ITO on glass (as labelled accordingly). All substrates show high transmittance in the visible range.

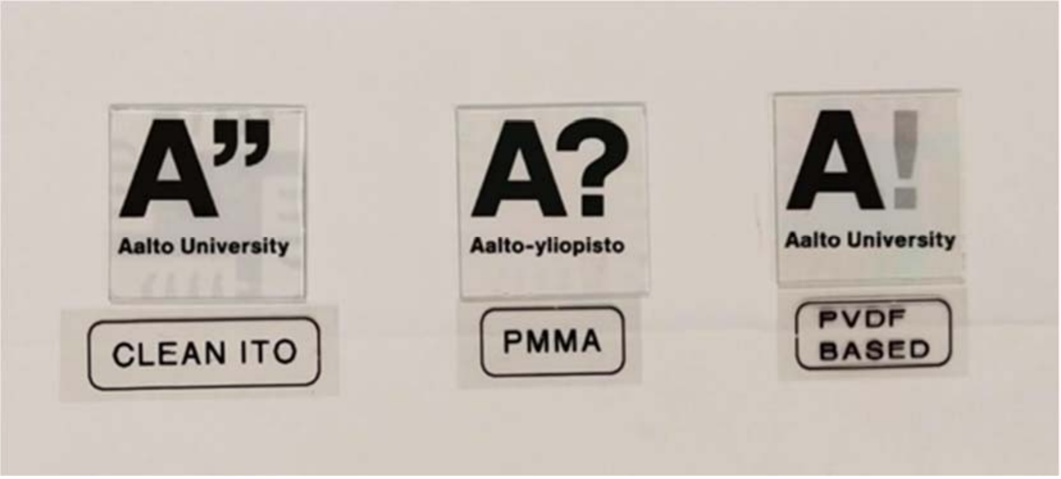

Figure S1. Optical image of different substrates used (as labelled accordingly).

3. Surface morphology of PVDF-based dielectric films

Figure S2 shows the AFM images (2 µm x 2 µm) of all the P(VDF)-based dielectric films, for the two different polymers (P(VDF-TrFE), P(VDF-TrFE-CFE)), two different solvents (cyclopentanone, methylethylketone) and different fabrication condition (spin-coating speeds) (Figure 1.a, main manuscript). All films present a *rice*-grain like structure with an approximate surface roughness (rms) lower than 3 - 4 nm.

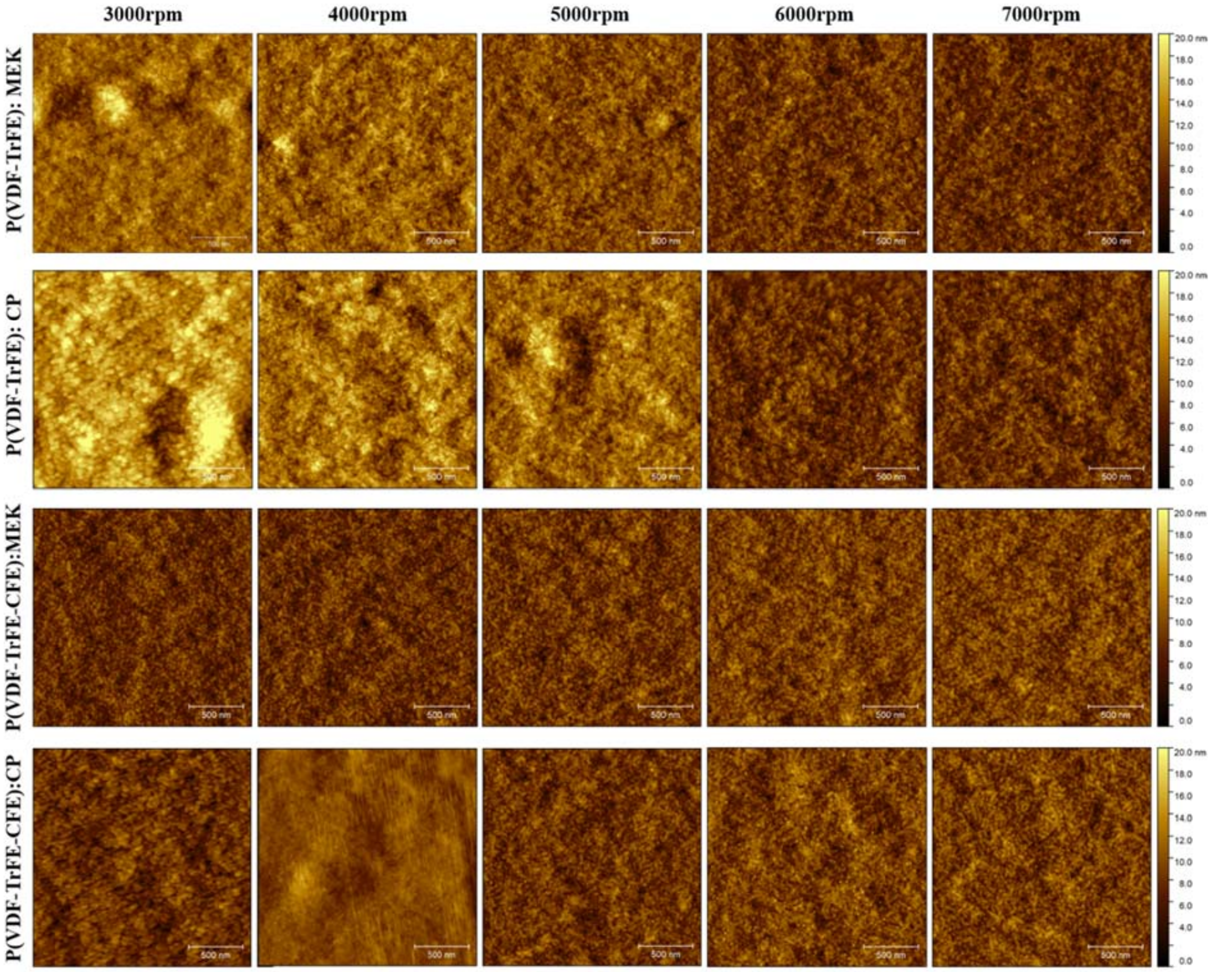

Figure S2. AFM images of the dielectric films for P(VDF)-based films with different polymers, solvents and spin-coating speeds. Column (left to right): 3000, 4000, 5000, 6000 and 7000 rpm, as labelled.

For comparison, an AFM image of a PMMA dielectric film is presented in Figure S3, which shows an extremely smooth surface with rms <1nm.

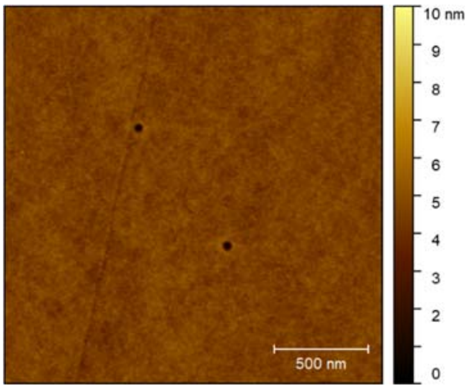

Figure S3. AFM image of PMMA surface.

4. Dielectric film properties: *C-f* measurement

Figure S4 shows the frequency dependence of the zero-bias (field) capacitance (C_0 = C (@V = 0V)) of the PVDF-based dielectric films. PMMA is also included as reference. The loss in the higher frequency region is closely related to the amount of amorphous region. Copolymer is typically more crystalline than terpolymer, thus losses occur at higher frequencies. Given its nature, no specific dependence is found for PMMA films.

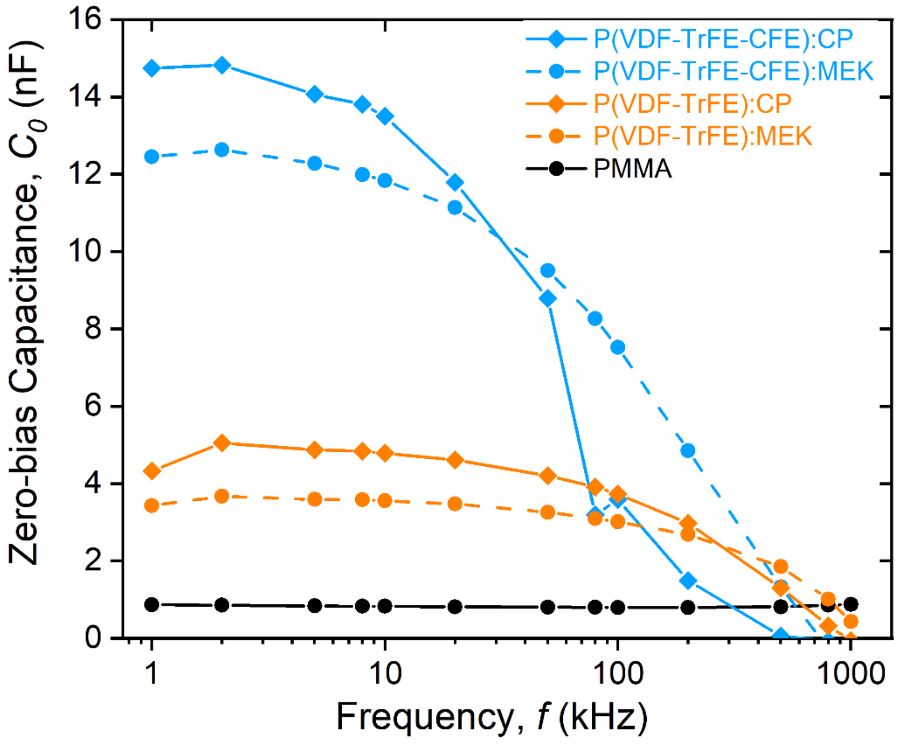

Figure S4. Frequency dependence of the zero-bias capacitance for PVDF-based films (PMMA is also included as reference).

5. Organic semiconductor characterization in single-layer OFET configuration

Figure S5 shows the transfer curves for (a) C8BTBT (45 nm) and (b) DFH-4T (45 nm) single layer organic field effect transistors. Devices were fabricated on ITO/PMMA substrates (on glass), in a BG-TC (bottom-gate/top-contacts) transistor configuration, with silver source and drain electrodes (70 nm). We found average values of mobilities of 6.3 (0.9) cm^2/Vs and threshold voltages of -45 V (+40 V) for holes and electrons, respectively.

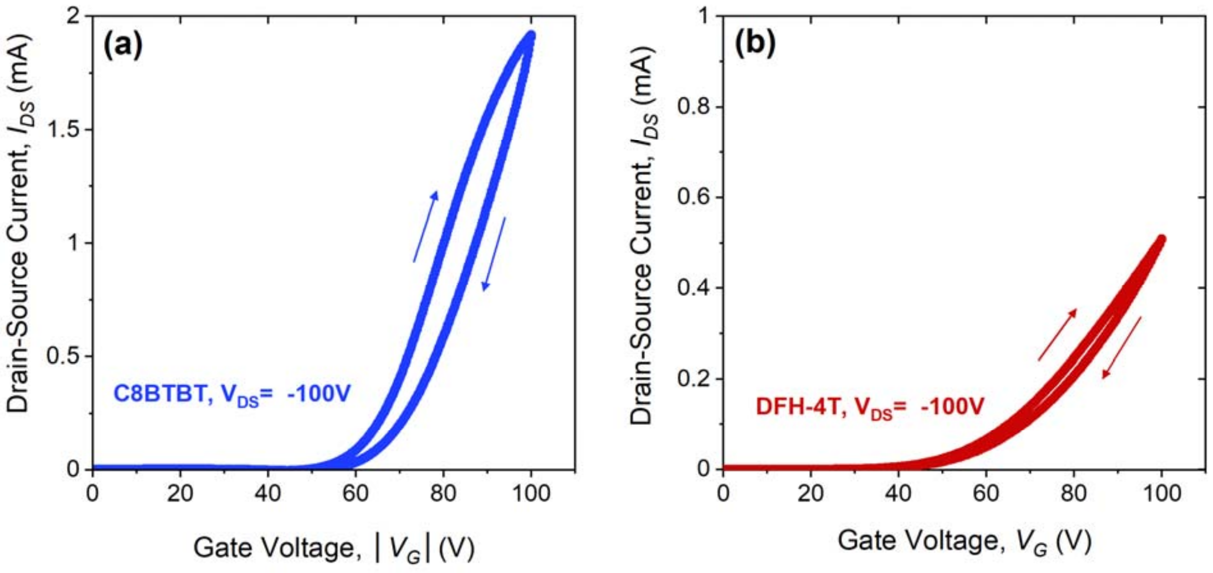

Figure S5. Transfer curves (I_{DS} *vs.* V_G) of single layer organic field-effect transistor based on (a) C8BTBT and (b) DFH-4T organic semiconductors. Both curves are measured with drain-source voltage, V_{DS} of |100| V. Arrows indicate the direction of the sweeps.
